# Assessment of Pulmonary Functions and Dysfunctions in Type II Diabetes Mellitus: A Comparative Cross-Sectional Study

**DOI:** 10.7759/cureus.35081

**Published:** 2023-02-16

**Authors:** Saumya Rajput, Rachna Parashar, Jai Prakash Sharma, Pragati Raghuwanshi, Abhijit P Pakhare, Rajnish Joshi, Sandip Hulke

**Affiliations:** 1 Department of Internal Medicine, All India Institute of Medical Sciences, Bhopal, Bhopal, IND; 2 Department of Physiology, All India Institute of Medical Sciences, Bhopal, Bhopal, IND; 3 Department of Anesthesiology, All India Institute of Medical Sciences, Bhopal, Bhopal, IND; 4 Department of Community and Family Medicine, All India Institute of Medical Sciences, Bhopal, Bhopal, IND

**Keywords:** hba1c variability, demographic profile, duration of diabetes, type 2 diabetes mellitus (dm), pulmonary functions

## Abstract

Background

Diabetes mellitus causes microvascular complications in the eyes and kidneys as well as the nervous system, among other parts of the body. Lungs are a potential target organ for diabetic microvascular complications and remain the least researched among diabetic patients. The aim of this study was to explore whether there is any difference in pulmonary functions in patients with diabetes mellitus compared to those without.

Methodology

A comparative cross-sectional study was conducted on 50 participants each with and without type II diabetes mellitus. Pulmonary function parameters, including forced vital capacity (FVC), forced expiratory volume in one second (FEV1), FEV1 as a percentage of FVC in percentage (FEV1%), peak expiratory flow rate in L/second (PEFR), forced expiratory flow rate in L/second in 25% of FVC (FEF25%), forced expiratory flow rate in L/second in 50% of FVC (FEF50%), forced expiratory flow rate in L/second in 75% of FVC (FEF75%), forced expiratory flow rate during 25-75% of expiration (FEF25-75%), and maximal voluntary ventilation (MVV), of both groups were analyzed using the NDD Large True Flow (Easy One) spirometer (NDD Meditechnik AG., Switzerland). A fully automated chemistry analyzer and linear chromatography were used for glycemic control measurements.

Results

All pulmonary function test parameter values were lower in participants with diabetes mellitus compared to those without, except FEV1% and PEFR, which indicates a mixed pattern of lung dysfunction. FVC had a significant negative correlation with the duration of diabetes (r = -0.299, p = 0.034).

Conclusions

Type II diabetes mellitus patients had significant dysfunction in pulmonary functions with early involvement of restrictive parameters which can be monitored/diagnosed by regularly following up patients by measuring pulmonary functions, and, hence, can be taken care of.

## Introduction

Type II diabetes mellitus (type II DM) is a metabolic disorder characterized by hyperglycemia and is caused by insulin resistance and a relative deficiency of insulin. Diabetes affected around 537 million adults (aged 20-79 years) in 2021. In developing countries, the majority of people with diabetes are in the age range of 45-64 years. The overall number of individuals living with diabetes is anticipated to reach 643 million by 2030 and 783 million by 2045. Three out of every four individuals with diabetes reside in low- and middle-income countries [1-3]. The burden of diabetes is especially high in Asian countries, including India, China, and Pakistan. There is also an alarming increase in comorbidities among Asians along with an increase in the incidence and prevalence of diabetes [4]. It is well known that type II DM is the leading cause of morbidity and mortality due to blindness, end-stage renal failure, non-traumatic limb amputations, and cardiovascular complications. The morbidities associated with diabetes mainly stem from macrovascular and microvascular complications [5]. Chronic hyperglycemia in diabetes results in the formation of advanced glycation end products (AGE). This induces pro-inflammatory changes and cross-linking of collagen in the endothelial basement membrane which leads to vascular complications. Microvascular disease-related complications are most profound in the retina, kidneys, and peripheral nerves [6]. Routine investigations are performed in diabetic patients not only to control the disease but also to evaluate for complications in these organs. Lungs, being a highly perfused vital organ, possess extensive microvasculature which remains unexplored, and lung involvement results in cardiovascular morbidities. These may be ignored due to the absence of early clinical signs [7]. Furthermore, if detected early, adequate therapy can be administered to sustain respiratory reserve and slow the onset of problems. Studies have described pulmonary function in type II DM patients with inconsistent results [8]. In the elderly, respiratory problems and diabetes are very common and frequently occur in the same patients. About 20% of people with chronic bronchitis or chronic obstructive pulmonary disease (COPD) also have diabetes, and nearly half of these patients also have concurrent metabolic syndrome [9]. Diabetes has been linked to COPD, although the opposite association has also been recorded. These two illnesses have lately been combined into a single generic condition known as *chronic systemic inflammatory syndrome* because they both exhibit low-grade chronic inflammation. In patients with asthma, abnormalities in glucose metabolism have also been identified [10]. Therefore, there is an urgent need to examine the pulmonary functions of type II DM patients to act as a future guide for the periodic evaluation of the same. It was hypothesized that the microvasculature of the lung should also undergo described pathophysiological changes and there will be some alterations in pulmonary functions of patients with type II DM. Pulmonary complications of DM have been poorly characterized and understood. Hence, periodic screening for pulmonary complications is not performed in the clinical scenario. However, the possibility of lung involvement, owing to the extensive microvascular circulation, cannot be ruled out. Therefore, this study aims to assess the alterations in pulmonary functions in type II DM in comparison to participants without DM and to explore whether there is a correlation between alterations in pulmonary functions, duration of disease, glycemic control, and demographic factors in type II DM.

## Materials and methods

Study setting and design

This comparative cross-sectional study was conducted in collaboration with the Diabetes Clinic and the Department of Physiology, All India Institute of Medical Sciences, Bhopal (AIIMS Bhopal).

Study population

The study was conducted on patients who were diagnosed with type II DM (study group) by treating clinicians as well as their relatives (control group) who visited the AIIMS Bhopal Diabetes Clinic. A total of 50 individuals with type II DM identified by the treating doctor were chosen at random from the patients attending the clinic between the ages of 40 and 64. Controls were chosen from the diabetics’ caretakers.

Exclusion criteria

Participants with a history of acute or chronic pulmonary disease, smoking, chronic illness, cardiorespiratory illness, anatomical abnormalities related to the thorax such as scoliosis or kyphosis, ascites, or any occupational exposure were excluded from the study.

Study procedure

Participants with type II DM who were between the ages of 40 and 64 were randomly selected. The sample size was 100, comprising 50 cases (previously diagnosed with type II DM) and 50 controls (non-diabetic relatives of type II DM patients). After obtaining written informed consent from both cases and controls, a detailed history was collected and a general examination was undertaken. All patients were given a questionnaire seeking thorough personal and medical background information.

Data collection

After recording the age (in years), sex, and duration of disease in both groups, the height was measured without shoes using a wall-mounted measuring tape. Weight was measured in kg using an electronic weighing machine after removing the shoes. Body mass index (BMI) was calculated as body weight (kg) divided by the square of height in meters (m^2^) according to the WHO guidelines.

Measurement of pulmonary function test

For the pulmonary function test (PFT), an NDD Large True Flow (EasyOne) spirometer (NDD Meditechnik AG., Switzerland) was used. PFT was performed at the Department of Physiology, AIIMS Bhopal. The subjects were made familiar with the instrument and the procedure for performing the test. All tests were conducted according to the American Thoracic Society/European Respiratory Society (ATS/ERS guidelines) in a quiet room by trained personnel [11]. The test was performed in a sitting position. The study participants were asked to take full inspiration which was followed by as much rapid and forceful expiration as possible in the mouthpiece. Three consecutive readings were recorded at an interval of 15 minutes, and the best reading among the three was selected for reproducibility and validity of the parameter. PFT parameters were considered acceptable if they fell within and between the maneuver acceptability criteria. Guidelines given in the joint statements on lung function testing of the ATS and the ERS were followed [12,13]. PFT parameters that were studied included forced vital capacity (FVC), forced expiratory volume in one second (FEV1), FEV1 as a percentage of FVC in percentage (FEV1%), peak expiratory flow rate in L/second (PEFR), forced expiratory flow rate in L/second in 25% of FVC (FEF25%), forced expiratory flow rate in L/second in 50% of FVC (FEF50%), forced expiratory flow rate in L/second in 75% of FVC (FEF75%), forced expiratory flow rate during 25-75% of expiration (FEF25-75%), and maximal voluntary ventilation (MVV). For all these parameters, the percentage of predicted values for the respective age, height, and weight were taken into consideration.

Measurement of glycemic control

Nearly 2 mL of venous blood was collected in an ethylenediamine tetraacetic acid vacutainer from all diabetic patients with aseptic precautions. Glycemic control was determined by recording fasting blood sugar (FBS), postprandial blood sugar (PPBS), and HbA1C levels. Blood sugar levels were obtained using the Random Access Fully Automated Chemistry Analyzer (Beckman Coulter Pvt. Ltd.), and HbA1c was measured by linear chromatography. All data were extracted and collected in a data extraction form and then transferred to an Excel sheet by independent data entry operators. Discrepant values were corrected by checking the data extraction form. Clean data were then analyzed statistically.

Statistical analysis

Data analysis was done using the EpiInfo^TM^ version 7 software. The questionnaire was created in the EpiInfo^TM^ software, and data were interpreted using Microsoft Office Excel 2007. For numerical variables, descriptive statistic measures such as mean and standard deviation were used for summarizing data. For categorical variables, frequency and percentage were used to summarize data. Differences between the mean values of PFT among cases and controls were tested using the unpaired t-test. The correlation between sociodemographic variables and glycemic control using different methods with each PFT variable was estimated by calculating Pearson’s correlation coefficient. Considering the small sample size, we did not perform a linear regression analysis to determine predictors of PFT. Statistical significance was set at p-values <0.05. We used R software with ggplot2, ggpairs, and ggstatsplot packages to create visualizations presented in the study [14].

Ethics and permissions

The study protocol was reviewed and approved by the Institutional Human Ethics Committee, AIIMS Bhopal (approval number: IHEC-LOP/2015/STS0059-2015). Every participant was provided a detailed patient information sheet explaining the study procedure, following which their queries, if any, were resolved. Subsequently, participants were enrolled after obtaining written informed consent.

## Results

**Figure 1:**
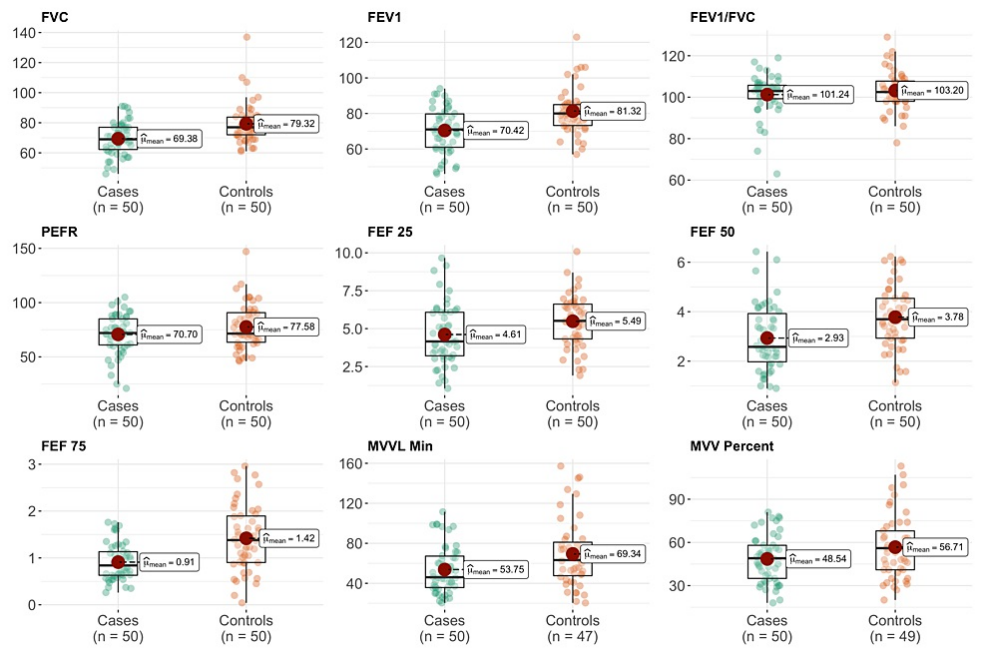
Variations in pulmonary function test parameters among type II diabetes mellitus (study group) and without diabetes (control group). FVC = force vital capacity; FEV1 = forced expiratory volume in one second; PEFR = peak expiratory flow rate; FEF = force expiratory flow; MVV = maximal voluntary ventilation

We enrolled 50 type II DM patients (mean age = 51.58 ± 7.49 years) and 50 participants without diabetes (mean age = 47.94 ± 5.98 years) in this study. The mean duration of diabetes was 6.9 ± 6.4 years. There were more females in both groups. The mean FVC (% predicted), FEV1 (% predicted), FEF25% (% predicted), FEF50% (% predicted), FEF75% (% predicted), FEF25-75% (% predicted), and MVV (% predicted) of the case group were found to be statistically significantly lower (p < 0.05) than that of the control group. However, the mean FEV1% (% predicted) and PEFR (% predicted) of the case group were found to be statistically insignificantly lower than that of the control group (Table 1, Figure 1). The mean values of FBS, PPBS, and HbA1C of diabetic patients (cases) were 143.58 ± 41.88 mg/dL, 229.66 ± 59.04 mg/dL, and 8.05 ± 1.35, respectively.

**Table 1:** Comparison of cases and controls on the basis of demographic characteristics and PFT parameters. *: significant (p < 0.05). BMI = body mass index; PFT = pulmonary function test; FVC = force vital capacity; FEV1 = forced expiratory volume in one second; PEFR = peak expiratory flow rate; FEF = force expiratory flow; MVV = maximal voluntary ventilation; % pred = % predicted

Variables	Cases - diabetes (n = 50)	Controls - no diabetes (n = 50)	P-value
	Mean (SD)		
Age (years)	51.58 (7.49)	47.94 (5.98)	0.0782
BMI (kg/m^2^)	25.64 (4.24)	23.58 (3.58)	0.0456
Gender (%)			
Male	5 (40)	15 (30)	0.0572
Female	45 (60)	35 (70)	0.0831
PFT parameter			
FVC (% pred)	69.38 (10.83)	79.32 (13.24)	0.0001*
FEV1 (% pred)	70.42 (12.59)	81.32 (12.64)	0.0001*
FEV1/FVC (% pred)	101.24 (9.78)	103.20 (9.30)	0.3070
PEFR (% pred)	70.70 (18.50)	77.58 (20.63)	0.0823
FEF25% (L/second)	4.61 (2.01)	5.49 (1.72)	0.0218*
FEF50% (L/second)	2.93 (1.32)	3.78 (1.26)	0.0015*
FEF75% (L/second)	0.91 (0.39)	1.42 (0.70)	0.0001*
FEF25-75% (%pred)	78.12 (25.47)	90.01 (31.59)	0.0409*
MVV (% pred)	48.54 (16.43)	57.36 (22.33)	0.0267*

**Figure 2:**
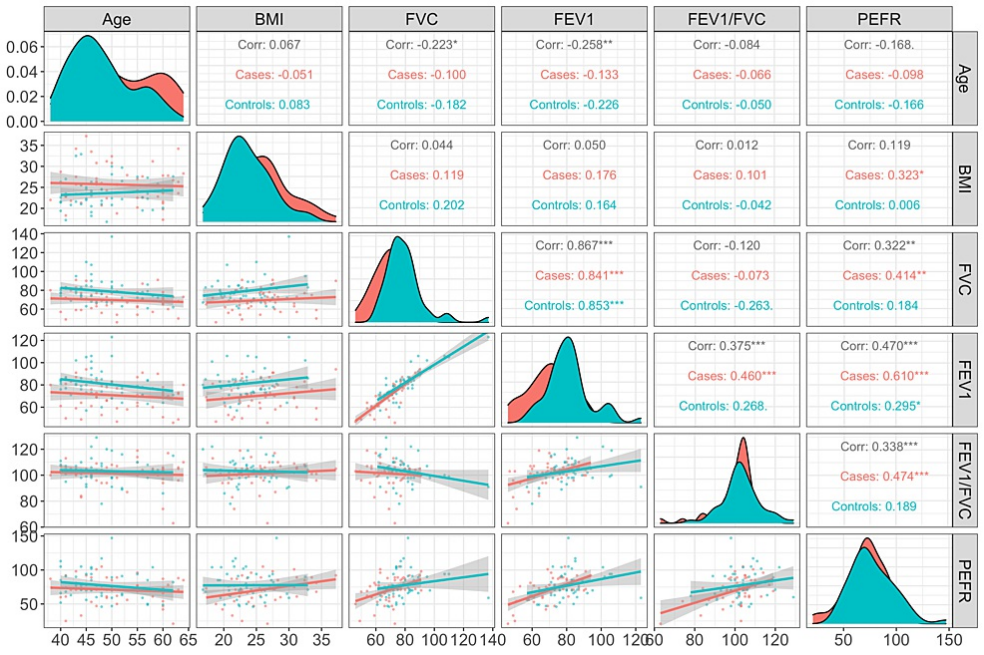
Scatterplots and correlation matrix for pulmonary function test among study participants. BMI = body mass index; FVC = forced vital capacity; FEV1 = forced expiratory volume in one second; PEFR = peak expiratory flow rate in L/second

**Figure 3:**
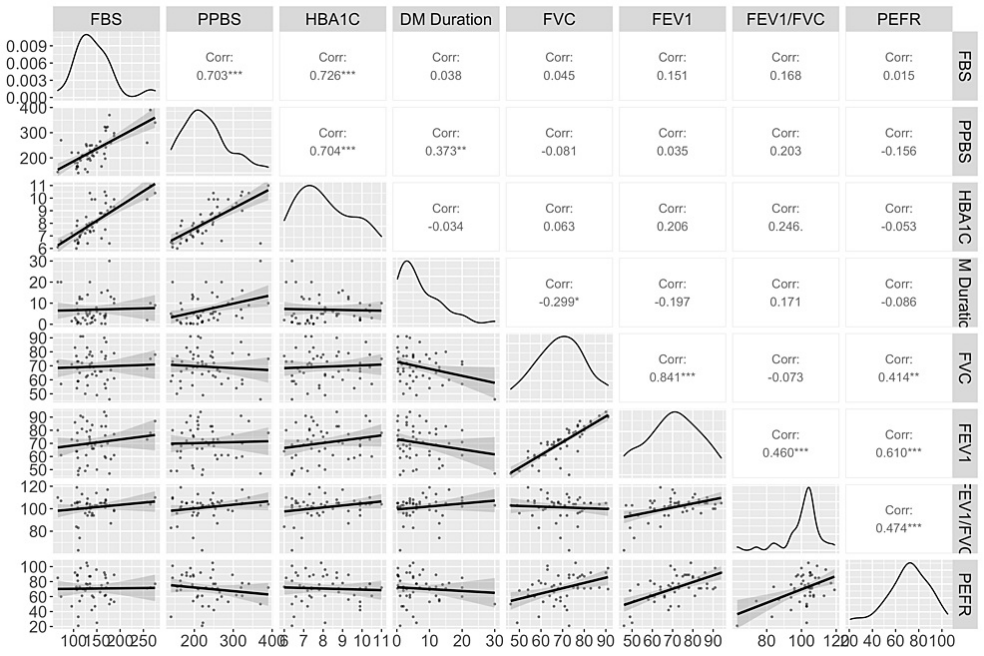
Scatterplots and correlation matrix for pulmonary function test and glycemic status among participants with diabetes mellitus. FBS = fasting blood sugar; PPBS = postprandial blood sugar; HbA1c = hemoglobin A1c; FVC = forced vital capacity; FEV1 = forced expiratory volume in one second; PEFR = peak expiratory flow rate in L/second

Disease duration was negatively correlated with FVC, and this relationship was statistically significant (p < 0.05). Age was negatively correlated with FVC; however, this relationship was not statistically significant (p > 0.05) among cases and controls. We did not find any specific correlation pattern among glycemic status variables such as FBS, PPBS, or HbA1C and PFT variables such as FVC, FEV1, FEV1/FVC ratio, or PEFR (Figure 2, Figure 3, Table 2).

**Table 2:** FVC (% pred), FEV1 (% pred), and FEV1/FVC (% pred) correlation with age, BMI, duration of disease, and glycemic control. BMI = body mass index; FBS = fasting blood sugar; PPBS = postprandial blood sugar; HbA1c = hemoglobin A1c; FVC = forced vital capacity; FEV1 = forced expiratory volume in one second; % pred = % predicted

Parameter		Correlation coefficient	
		R	P-value
FVC (% pred)			
Age	Cases	-0.1005	0.4876
	Controls	-0.1817	0.2066
BMI (kg/m^2^)	Cases	0.1194	0.4087
	Controls	0.2024	0.1587
Duration of disease		-0.2993	0.0347
Glycaemic control			
FBS (mg/dL)		0.0449	0.7570
PPBS (mg/dL)		-0.0815	0.5739
HbA1C (mg/dL)		0.0627	0.6652
FEV1 (% pred)			
Age	Cases	-0.1334	0.3558
	Controls	-0.2264	0.1138
BMI (kg/m^2^)	Cases	0.1760	0.2215
	Controls	0.1643	0.2542
Duration of disease		-0.1969	0.1706
Glycemic control			
FBS (mg/dL)		0.1509	0.2955
PPBS (mg/dL)		0.0350	0.8095
HbA1C (mg/dL)		0.2063	0.1505
FEV1/FVC (% pred)			
Age	Cases	-0.0657	0.6501
	Controls	-0.0504	0.7820
BMI (kg/m^2^)	Cases	0.1012	0.4843
	Controls	-0.0419	0.7728
Duration of disease		0.1712	0.2345
Glycemic control			
FBS (mg/dL)		0.1683	0.2427
PPBS (mg/dL)		0.2026	0.1582
HbA1C (mg/dL)		0.2459	0.0851

A statistically significant negative correlation was observed between MVV (% predicted) and the age of the case group only. No other statistically significant relationship was noted between MVV (% predicted) and demographic factors or glycemic control parameters (Table 3).

**Table 3:** PEFR (% pred) and MVV (% pred) correlation with age, BMI, duration of disease, and glycemic control. BMI = body mass index; FBS = fasting blood sugar; PPBS = postprandial blood sugar; HbA1c = hemoglobin A1c; PEFR = peak expiratory flow rate; MVV = maximal voluntary ventilation; % pred = % predicted

Parameter		Correlation coefficient	
		R	P-value
PEFR (% pred)			
Age	Cases	-0.0980	0.4983
	Controls	-0.1658	0.2499
BMI (kg/m^2^)	Cases	0.3233	0.0220
	Controls	0.0064	0.9648
Duration of disease		-0.0863	0.5511
Glycemic control			
FBS (mg/dL)		0.0152	0.9163
PPBS (mg/dL)		-0.1563	0.2785
HbA1C (mg/dL)		-0.0534	0.7125
MVV (% pred)			
Age	Cases	-0.3333	0.0180
	Controls	-0.2370	0.0975
BMI (kg/m^2^)	Cases	0.2212	0.1226
	Controls	0.2482	0.0822
Duration of disease		-0.0773	0.5936
Glycemic control			
FBS (mg/dL)		0.0786	0.5873
PPBS (mg/dL)		0.0309	0.8314
HbA1C (mg/dL)		-0.0122	0.9329

Age was negatively correlated with FEF50%, and this relationship was statistically significant. Glycemic control was also negatively correlated with FEF50%, but this relationship was not statistically significant. A statistically significant negative correlation existed between the age of both groups and FEF75%. No statistically significant relationship existed between FEF25-75% and demographic factors or glycemic control parameters (Table 4).

**Table 4:** The correlation of FEF25%, FEF50%, FEF75%, and FEF25-75% (L/second) with age, BMI, duration of disease, and glycemic control. BMI = body mass index; FBS = fasting blood sugar; PPBS = postprandial blood sugar; HbA1c = hemoglobin A1c; FEF = forced expiratory flow

Parameter		Correlation coefficient	
		R	P-value
FEF25%			
Age	Cases	-0.2527	0.0766
	Controls	-0.3442	0.0144
BMI (kg/m^2^)	Cases	0.2168	0.1304
	Controls	-0.0986	0.4959
Duration of disease		-0.0144	0.9211
Glycemic control			
FBS (mg/dL)		-0.1905	0.1852
PPBS (mg/dL)		-0.2067	0.1497
HbA1C (mg/dL)		-0.2164	0.1311
FEF50%			
Age	Cases	-0.2912	0.0402
	Controls	-0.3561	0.0111
BMI (kg/m^2^)	Cases	0.2977	0.0357
	Controls	-0.0209	0.8856
Duration of disease		-0.0418	0.7729
Glycemic control			
FBS (mg/dL)		-0.1263	0.3822
PPBS (mg/dL)		-0.1103	0.4456
HbA1C (mg/dL)		-0.0604	0.6768
FEF75%			
Age	Cases	-0.4826	0.0004
	Controls	-0.3357	0.0172
BMI (kg/m^2^)	Cases	0.0268	0.8536
	Controls	-0.1025	0.4786
Duration of disease		0.0384	0.7911
Glycemic control			
FBS (mg/dL)		-0.0014	0.9925
PPBS (mg/dL)		-0.0423	0.7707
HbA1C (mg/dL)		-0.0035	0.9805
FEV (25-75%)			
Age	Cases	-0.0485	0.7378
	Controls	-0.1049	0.4683
BMI (kg/m^2^)	Cases	0.2434	0.0886
	Controls	0.1109	0.4432
Duration of disease		0.0033	0.9820
Glycemic control			
FBS (mg/dL)		-0.0085	0.9532
PPBS (mg/dL)		0.0210	0.8849
HbA1C (mg/dL)		0.1265	0.3814

## Discussion

The aim of this study was to assess the pulmonary functions of type II DM patients compared to healthy controls. We hypothesized that PFT parameters would be altered in type II DM patients and the values would be lower than those of participants without diabetes. In the present study, pulmonary function assessment showed that some spirometry values (FVC, FEV1, FEF25%, FEF50%, FEF75%, FEF25-75%, and MVV) were significantly lower (p < 0.05) in type II DM patients compared to controls. On the other hand, FEV1% and PEFR were not significantly reduced. However, FEV1% was within the normal range, reflecting that both FEV1 and FVC were affected in DM in relatively the same proportion so that the ratio did not deviate from the normal. Our hypothesis was supported up to a certain degree by Irfan et al. [15].

The ratio of FEV1/FVC was preserved, and the reduction in values of both FVC and FEV was noted. The below-normal percentage predicted values indicate a mixed pattern of lung dysfunction (restrictive along with obstructive). This is consistent with studies by Panpalia et al. and Shah et al. [16,17], who also deduced a mixed nature of involvement. Hyperglycemia in DM is associated with the increased formation of AGEs. AGEs cause lung dysfunction through their pro-inflammatory effect, suggesting obstructive lung mechanical dysfunction. The second mechanism is AGE-induced cross-linking in the connective tissue of the lung, suggestive of restrictive lung mechanical dysfunction in diabetes.

MVV is the maximum breathing capacity that is affected by poor respiratory muscle strength. A statistically significant reduction in MVV values in type II DM patients compared to controls may be due to poor skeletal muscle strength caused by increased protein catabolism in type II DM. Some studies have reported similar results as our study, while others have reported contradictory results in some parameters [18,19]. Several studies have reported significant reductions in PEFR in type II DM patients compared to those without DM, which is contradictory to our findings [20-22].

Some studies have also reported insignificant differences in spirometric PFTs between patients with diabetes and normal control subjects [23,24]. The possible reasons for such disparities may be differences in race, age group, duration, and glycemic control of diabetes in the studied population [25].

The other objective of our study was to determine the correlation of PFT parameters with age, BMI, duration of disease, and glycemic control. We found a statistically significant negative correlation of age with FEF50%, FEF75%, and MVV. Other parameters did not show any significant correlation with age, which may be understood by early autonomic neuropathic changes in the lung, as suggested by Williams et al. and Parashar et al. [26,27]. Duration of disease was negatively correlated with FVC, and this relationship was statistically significant. Furthermore, FVC is considered a marker for restrictive pattern disease [28].

The mean duration of diabetes in this study was 6.9 ± 6.4 years. The longer duration of diabetes exposes the patient to an increased risk of microvascular damage due to AGEs. However, in the present study, the duration of the disease did not appear to predict lung damage, except for FVC. Further, it may suggest that FVC, being the most sensitive, is affected earlier in patients with type II DM. Glycemic control was not observed to be associated with PFT parameters. This result was consistent with some previous studies [29,30]. Thus, glycemic control, as determined by FBS, PPBS, and glycosylated hemoglobin levels, was not a significant determinant of lung function in diabetes.

Limitations of the study

The study had some limitations. First, the sample size was small. We recommend further multicenter studies with a larger sample size. Second, this was a hospital-based study where patients were expected to have more comorbidities, which may have impacted the results. Third, we recruited healthy controls from relatives of the patients. Because relatives might share genetic and environmental exposures, it is likely to bias the study results toward the null hypothesis. Further, this was a comparative cross-sectional study and did not consider the decline from the onset of diabetes, which can be investigated in a longitudinal study design. Moreover, PFT was done at just one point in time which is likely to be biased due to measurement errors.

## Conclusions

Lung functions are likely to be reduced in individuals with type II DM. Mostly, lung dysfunction is mixed in nature (obstructive or restrictive pattern). Because most participants did not have any symptoms related to deranged PFT, we need to follow up if symptoms appear in the long run. We may need to initiate lung-protective strategies in individuals with DM, such as tight control of sugars, tobacco cessation, minimal exposure to air pollutants, and occupational exposure which may add to pulmonary insult, along with close supervision of lung functions in type II DM patients. Likewise, avoidance of drugs that have a potential for chronic lung injury, such as immunosuppressants, may be advocated.
